# Post-Visit Patient Understanding About Newly Prescribed Medications

**DOI:** 10.1007/s11606-020-06540-4

**Published:** 2021-02-05

**Authors:** Timothy Ho, Blanca S. Campos, Derjung M. Tarn

**Affiliations:** Department of Family Medicine, David Geffen School of Medicine at UCLA, University of California, Los Angeles, Los Angeles, CA USA

**Keywords:** physician-patient relations, prescription medications, mixed methods research, adverse effects

## Abstract

**Background:**

Good patient understanding of basic medication-related information such as directions for use and side effects promotes medication adherence, but information is lacking about how well patients understand basic medication-related information after their office visits.

**Objective:**

The purpose of this study is to investigate post-visit patient understanding about newly prescribed medications.

**Design:**

Secondary mixed methods analysis comparing patient survey responses about newly prescribed medications to information conveyed by physicians during office visits (from audio recordings of office visits).

**Participants:**

Eighty-one patients aged 50 and older who discussed newly prescribed medications during an outpatient office visit.

**Main Measures:**

Accurate patient identification of medication dose, number of pills, frequency of use, duration of use, and potential side effects.

**Key Results:**

The 81 patients in this study received 111 newly prescribed medications. For over 70% of all newly prescribed medications, patients correctly identified the number of pills, frequency of use, duration of use, and dose, regardless of whether the physician mentioned the information during the office visit. However, for 34 of 62 medications (55%) for which side effects were not conveyed and 11 of 49 medications (22%) for which physicians discussed side effects, patients reported that the medication lacked side effects. Analysis of transcribed office visits showed that potential reasons for this finding included failure of physicians to mention or to use the term “side effects” during visits, the prescription of multiple medications during the visit, and lack of patient engagement in the conversation.

**Conclusions:**

Many patients correctly identified information related to directions for taking a newly prescribed medication, even without physician counseling, but when physicians failed to convey potential medication side effects, many assumed that a medication had no side effects. It may be sufficient for physicians to provide written information about medication directions and dosing, and tailor their limited time to discussing medication side effects.

## INTRODUCTION

Poor patient understanding about the benefits and risks of a prescribed medication or directions for use serves as a barrier to medication adherence.^1^ Medication nonadherence in turn results in adverse health outcomes such as increased hospitalizations and mortality, particularly in older patients.^2^ Poor physician-patient communication may contribute to poor patient understanding and subsequent medication nonadherence.^3^ Previous studies have demonstrated suboptimal physician communication regarding basic information about medications, including directions for use and side effects.^4, 5^ In fact, physicians often do not mention the types of side effects that interest patients.^6^ A handful of studies have examined patient recall of medication-related information that physicians convey during office visits, but these studies included mostly younger patients, and did not assess potential reasons for poor patient understanding of what physicians discussed.^7, 8^

This study aims to address gaps in the literature regarding post-visit understanding about newly prescribed medications in patients aged 50 and older. The goals of this study were to (1) determine post-visit understanding of basic information (i.e., directions for use, side effects) about newly prescribed medications in patients aged 50 and older, and (2) evaluate potential reasons for poor patient understanding of information conveyed by physicians.

## METHODS

This is a secondary analysis of mixed methods (qualitative and quantitative) data collected in 2009–2010 for a randomized clinical trial of an intervention to improve communication about newly prescribed medications.^9^ The purpose of this analysis was not to examine the effect of the intervention (which sought to promote communication about newly prescribed medications), but to assess patient understanding of specific topics of information that physicians conveyed. We analyzed both audio recordings of office visits and subsequent survey data from the original study, and used qualitative analyses of what actually transpired during the office visits to investigate potential reasons for some of our quantitative findings. In the original study, physicians were randomized to participate in an educational session to prompt communication about basic information concerning newly prescribed medications (medication name, directions for use, duration of use, and side effects). The study measured whether they communicated these pieces of information to patients during office visits. No patient randomization occurred. Patients were recruited from an academic medical center and provided informed consent. The study recruited 256 English-speaking primary care patients aged 50 years and older who had a new, worsening, or uncontrolled medical problem. Data collected included immediate post-visit patient surveys and audio recordings of office visits. Surveys asked patients to write-in information about their newly prescribed medication: number of pills; frequency of use; duration of use; dose; and possible side effects. Patients were allowed to refer to their prescriptions or written information provided at the end of the visit to complete the survey. Other survey items queried patients about their demographics and health literacy.^10^

This study analyzed data from 81 patients in the original study who were newly prescribed a medication during their office visit and discussed the prescription with their provider. Qualitative content analysis of transcribed office visit audio recordings was previously performed to assess physician transmission of information regarding a newly prescribed medication’s directions for use (number of pills, frequency of use, duration of use, dosing, and potential side effects).^9^ For this study, we first examined open-ended survey responses to determine whether or not patients correctly identified each medication-related element (yes/no) based on whether or not the physician mentioned the medication-related element in the office visit. We also assessed the range of patient responses when they did not correctly identify the requested information. For each medication-related element, missing survey responses were excluded from the analyses. We excluded creams from our analysis of the number of pills taken.

For medication side effects, we performed additional thematic analyses of the transcripts to examine instances when physicians commented on a medication’s side effects during the encounter, but patients indicated that the medication lacked side effects. Analyses focused on examining characteristics of conversations that may have contributed to these patient perceptions. One investigator (BSC) performed initial categorizations. The other two investigators (TH and DMT) worked together to verify the initial analysis; discrepancies were resolved via consensus. The study protocol was approved by the UCLA Institutional Review Board.

## RESULTS

The 81 patients in this study were newly prescribed a total of 111 medications. Patients were mostly women (*n* = 49 [60.5%]), had a mean age of 60.4 years (SD = 8.1), and 66 (81%) had at least some college education (Table 1). Patients reported taking an average of 6.7 (SD = 3.9) medications and dietary supplements.

**Table 1:** Characteristics of Patients Newly Prescribed Medication; *n* = 81

Characteristic	*n* (%) or mean (SD)
Age, mean (SD; range)	60.4 (8.1; 50–91)
Female, *n* (%)	49 (60.5)
Education, *n* (%)	
High school or less	14 (17.5)
Some college	28 (35)
College graduate	38 (47.5)
Race/ethnicity, *n* (%)	
White	48 (59.3)
African American	15 (18.5)
Hispanic	12 (14.8)
Asian	5 (6.2)
Other	1 (1.2)
Health literacy score, mean (SD)*	6.8 (0.49)

*Health literacy score ranges from 0–7, with higher scores indicating greater health literacy^10^

**Figure 1:**
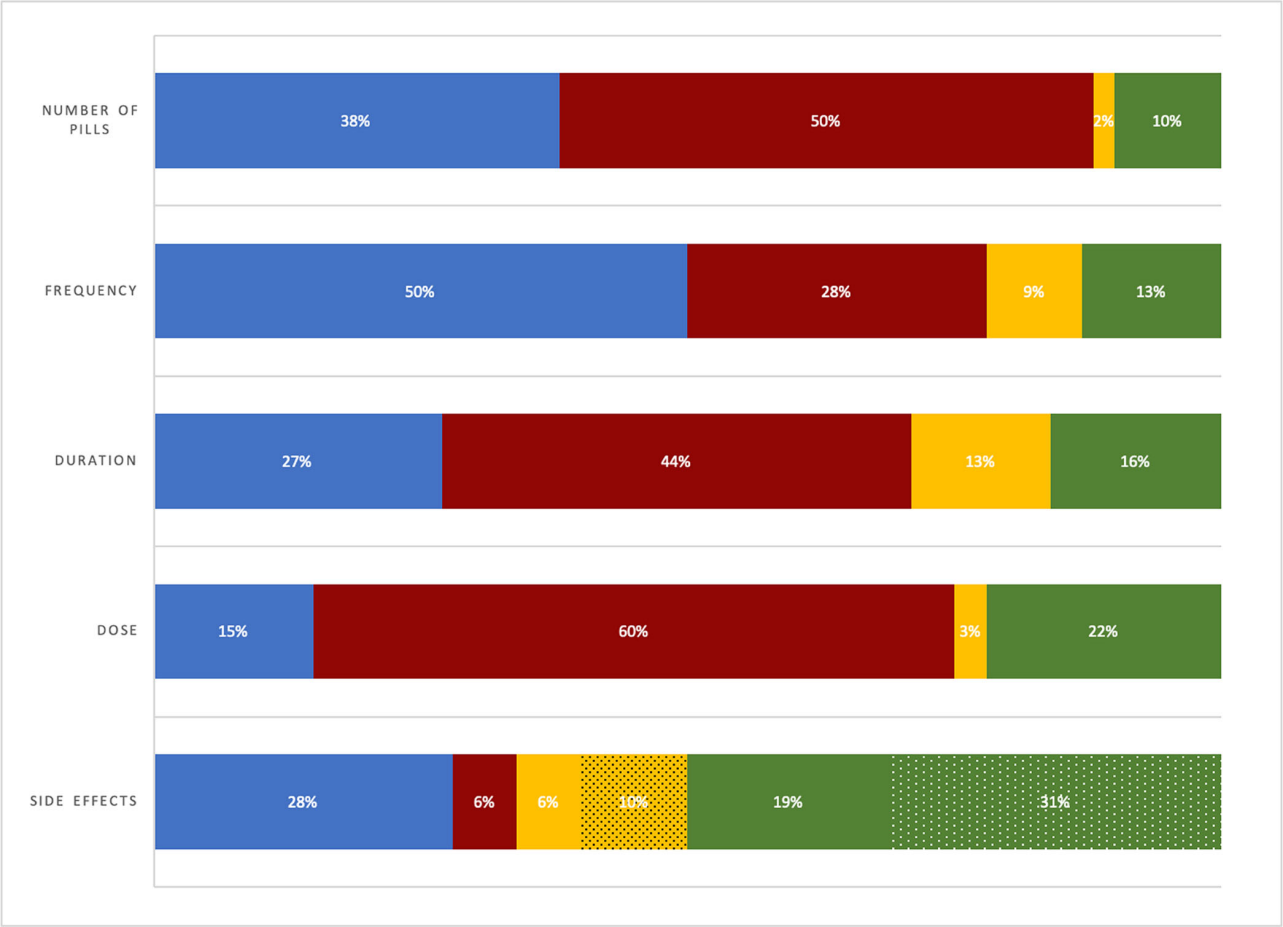
Frequency with which patients recalled information regarding newly prescribed medications immediately post-visit, by whether physicians mentioned the information during the office visit, *n* = 111 medications. Blue bars indicate accurate patient report of physician discussion; red bars indicate accurate patient report but lack of physician discussion; yellow bars indicate inaccurate patient report of physician discussion; and green bars indicate inaccurate patient report with lack of physician discussion. Dotted bars for side effects indicate that the patient reported the medication had “no side effects.”

Physicians conveyed information about medication number of pills, duration of use, and dose for 40%, 40%, and 18% of all newly prescribed medications, respectively. Side effects were mentioned for 44% of medications, and frequency of use for 59%. For over 70% of all prescribed medications, patients accurately identified information about the number of pills, frequency of use, duration of use, and medication dose, regardless of whether the physician mentioned the information during the office visit. All patients who lacked knowledge about the requested information regarding number of pills, frequency of use, duration of use, and medication dose wrote comments indicating “?;” “don’t know;” or “N/A.” Of 62 medications for which physicians made no mention of side effects, patients indicated that no side effects existed for 34 (55%; 31% of total medications). Patient responses regarding a perceived lack of side effects included “none;” “no side effect;” or “N/A.” Of 49 medications for which physicians conveyed side effects, patients indicated that no side effects existed for 11 (23%; 10% of total medications) (Figure 1).

Table 2 depicts characteristics of physician-patient conversations that may have led patients to believe that a medication lacked side effects. Common reasons included poor patient engagement in conversations regarding medications (*n* = 20; 44.4%) and lack of physician mention of the term “side effects” (*n* = 7; 15.6%). For example, a physician describing the side effects of Motrin told a patient: “you’ve got to be careful not to affect your kidneys too much” without explicitly mentioning the term “side effect.” Other physicians stated that medications were “generally well tolerated” or had “virtually” no side effects (*n* = 5; 11.1%).

**Table 2:** Characteristics of Physician-Patient Interactions Associated with Patient Beliefs that a Newly Prescribed Medication Lacked Side Effects; *n* = 45 medications prescribed to 39 patients

Characteristic of physician-patient interaction	*n* (%)
Physician did not convey potential side effects	35 (77.8%)
Patient did not engage in conversation about side effect (e.g., no follow-up comment)	20 (44.4%)
Multiple medications were prescribed during the office visit	21 (46.7%)
Physician did not explicitly use the word “side effect”	7 (15.6%)
Physician qualified discussion of side effects with “virtually none” or “generally well tolerated”	5 (11.1%)
Physician used complex medical terminology	1 (2.2%)
Patient told physician that they would not get side effect	1 (2.2%)

## DISCUSSION

This study demonstrates that patients may be able to ascertain basic information regarding medication directions regardless of whether a physician verbally conveys the information during an office visit, but understanding regarding the side effects of newly prescribed medications may be subject to the content of physician counseling. When physicians did not mention the possibility of a medication side effect, 55% of patients reported that the medication had no side effects. The words that physicians use seem to matter. For 15.6% of medications for which physicians discussed side effects but did not explicitly use the term “side effect,” patients reported that the medication had no side effects. Yet our study found that at least 70% of patients accurately reported on medication dosing and directions for use. These recall rates are much higher than found in a previous study of patients older than 40, in which patients recalled approximately one-third of each of the medication-related pieces of information provided.^8^ We speculate that this higher than expected rate of accurate reporting resulted from patient use of written information or prescriptions in hand when completing the surveys. Taken together, our findings suggest that physicians may not need to spend much time communicating about information that can be found on patient prescriptions or after-visit summaries (e.g., medication dose, number of pills, frequency of use), but could consider spending their limited time discussing medication side effects.

This investigation of recall and understanding builds on previous studies using similar methods to examine patient understanding of medication-related information, by focusing on a group of patients aged 50 and older.^7, 8^ While this study found that patients accurately reported 35–83% of medication-related information about newly prescribed medications, previous studies found that patients who were predominantly younger had higher recall rates of 83–90%.^7^ These findings highlight the added importance of ensuring understanding when prescribing medications for older patients, such as through the use of the “teach-back” method, where patients are asked to explain information in their own words.^11^

The study had several limitations. Over half of the patients had at least some college education and high levels of health literacy. Understanding about prescriptions may have been enhanced because patients were allowed to use written information to respond to survey questions, but we did not collect data on how often patients used written information to assist with survey completion.

In summary, this study highlights the importance of the words that physicians use when counseling patients about the potential side effects of newly prescribed medications. Specifically, when physicians do not mention the possibility of medication side effects or fail to explicitly use the term “side effects,” patients may believe that a medication has no side effects. Many older patients worry about side effects when starting a newly prescribed medication.^12^ Those who leave an office visit under the assumption that a medication has no side effects may be at greater risk for future medication nonadherence when they are later informed about potential adverse events.
